# The Animal-Human Interface in Farm Animal Production: Animal Fear, Stress, Reproduction and Welfare

**DOI:** 10.3390/ani12040487

**Published:** 2022-02-16

**Authors:** Rutu Y. Acharya, Paul H. Hemsworth, Grahame J. Coleman, James E. Kinder

**Affiliations:** 1 The Animal Welfare Science Centre, Faculty of Veterinary and Agricultural Sciences, The University of Melbourne, Parkville, VIC 3010, Australia; phh@unimelb.edu.au (P.H.H.); grahame.coleman@unimelb.edu.au (G.J.C.); 2 Department of Animal Sciences, The Ohio State University, Columbus, OH 43210, USA; kinder.15@osu.edu

**Keywords:** human-animal interactions, stress, neuroendocrinology, animal welfare, reproduction, productivity

## Abstract

**Simple Summary:**

For at least the last four decades, the focus of animal welfare research, quality assurance, and policy initiatives has been on measuring behavioural and physiological stress responses in animals. In the last decade, however, this focus of animal welfare research has shifted to the consequences of these behavioural and physiological stress responses rather than only the responses per se. Modern-day farming, even with the intensification and automation requires regular monitoring and interactions by stockpeople. Research conducted in both experimental and commercial settings has shown widespread effects of the human-animal interactions on behaviour, physiology, and reproductive performance in farm animals. In this paper, we review the implications of human-animal interactions on reproduction in farm animals.

**Abstract:**

A negative human-animal relationship (HAR) from the perspective of the animal is a limiting factor affecting farm animal welfare, as well as farm animal productivity. Research in farm animals has elucidated sequential relationships between stockperson attitudes, stockperson behaviour, farm animal fear behaviour, farm animal stress physiology, and farm animal productivity. In situations where stockperson attitudes to and interactions with farm animals are sub-optimal, through animal fear and stress, both animal welfare and productivity, including reproductive performance, can be compromised. There is a growing body of evidence that farm animals often seek and enjoy interacting with humans, but our understanding of the effects of a positive HAR on stress resilience and productivity in farm animals is limited. In this review, we explore the pathways by which stress induced by human-animal interactions can negatively affect farm animal reproduction, in particular, via inhibitory effects on the secretion of gonadotrophins. We also review the current knowledge of the stockperson characteristics and the nature of stockperson interactions that affect fear and physiological stress in farm animals. The contents of this review provide an insight into the importance of the HAR on farm animal welfare and reproduction while highlighting the gap in knowledge regarding the effects of a positive HAR on farm animals.

## 1. Introduction

The structure of modern livestock farming has generally evolved in the last seven decades from small family-owned enterprises to large-scale intensive commercial systems to meet the growing need for economically affordable and safe meat, milk, and eggs [1,2,3]. This intensification of farming involving concentration on fewer farms with increased animal numbers but with a decreased workforce has resulted in each stockperson managing more animals with many of the stockperson interactions such as restraint, vaccination, and surgical interventions tending towards negative interactions with animals with reduced time or effort allocated to positive interactions [4,5,6].

The modern-day management of animals by humans from household pets to livestock is based on two important principles: management practices that comply with the objectives of human profit, benefits, or pleasure; and alternatively, management practices to comply with humane care of animals [7]. The latter is based on the widely perceived view that the use of animals by humans is acceptable provided that such use is humane. While not always sufficiently recognised, human-animal interactions, both direct and indirect, are an inevitable feature of livestock production and the interactions at this animal-human interface can affect farm animal welfare and productivity [8]. Animal management on farms includes human resource management practices, such as employee selection and training, and animal management practices, such as best practices in housing and husbandry, obviously affect farm animal welfare and productivity. A number of stockperson characteristics and environmental factors affect farm animal welfare and productivity. According to Blumberg and Pringle’s [9] model of employee work performance, the three main classes of contributing factors that affect a person’s performance are: capacity, willingness, and opportunity. These three characteristics are useful when considering the essential but often the little-appreciated contribution of the stockperson to farm animal welfare and productivity: capacity includes variables such as skills, health, ability, and knowledge; willingness includes motivation, job satisfaction, attitude to the animals, and work attitude; and opportunity includes working conditions, actions of co-workers and organisational policies and procedures [5,10].

In managing livestock in both extensive and intensive settings, stockpeople interact regularly with the animals in their care and all these interactions by humans have implications on future responses of livestock animals to humans. The human-animal relationship (HAR) can be conceptualized in terms of inter-individual relationships which are based on the history of regular interactions between the two individuals and thus this history of interactions influences each individual partner’s perception of the relationship and in turn, guides future interactions [5,11]. As we will consider later in this review, results from HAR-focused studies indicate through conditioning, the animal’s behavioural responses to humans are regulated by the nature of previous experiences contiguous with the time of interactions with humans leading to a stimulus-specific conditioned response to humans [12].

Many human-animal interactions in the livestock industries are associated with regular welfare monitoring by stockpeople which often involve only visual contact between the stockperson and the animals, perhaps without the stockperson entering the pen or paddock where the animals are housed or confined. Most farm animals need to be moved at times and visual interactions, such as waving, speed of movement and positioning, and auditory interactions, such as talking, shouting, whistling, and clapping, as well as artificial noise, such as shaking rattles and banging implements on solid surfaces, may be used [5]. In some industries, tactile interactions with the hand or a handling implement, such as poly pipe or electric goad, may be used. Some of these human interactions have been shown to be fear-provoking for the animals.

Human-animal interactions also occur in situations in which animals must be restrained and subjected to management or health procedures. Some farm animals are rarely restrained during their lives, while others are restrained on a regular basis. Some sort of restraint is used for weighing, milking, vaccinating, and blood sampling, and animals are restrained for procedures that are painful such as castration, branding, ear tagging, and de-horning [5]. It may be possible to reduce or eliminate some of these procedures. Procedures, such as vaccinations and blood sampling for diagnosis, are necessary to improve the health and thus the welfare of the animals and to some extent discomfort or pain is justified in the welfare interests of the animal. Procedures such as milking and shearing, are directly related to the reason the animals are utilised by humans and could only be eliminated if the industry is no longer practical for milk or wool production purposes. Weighing, ear tagging, castration, and de-horning are justified by facilitating management, improving product quality, and/or reducing the possibility of injury to animals or humans. The association of fear and pain with these husbandry procedures conducted by humans performing these practices will increase the fear of humans which animals express in other situations, such as during routine inspections. The effect these procedures have on the fear response of the animals to humans relates both to the aversiveness of the procedure, and the association by the animal of humans with that aversion. Rushen and colleagues [13] have provided convincing evidence that performing an aversive treatment at a specific location or by either an unfamiliar or familiar stockperson wearing different distinctive clothing may prevent farm animals from associating the procedure with a particular stockperson.

All of these stockperson interactions, therefore, contribute to the overall relationship that animals have with humans and are important in determining if the relationship from the animal’s perspective is positive, neutral, or negative. While Hemsworth, Coleman, and colleagues [5,10,12] have reviewed the human factors affecting stockperson interactions with farm animals, the present review will focus on the implications of stockperson behaviour on animal reproduction and the behavioural and physiological mechanisms whereby these interactions can affect reproductive outcomes.

## 2. Emotions Associated with the Responses of Animals to Humans

There is evidence that farm animals can be highly fearful of humans, but farm animals can also seek and enjoy interactions with humans. To understand these varying responses of farm animals to humans, we will utilise firstly a comprehensive and empirically well-supported model of mammalian behavioural control by Panksepp and colleagues [14,15,16,17] and secondly a perspective on this model by Mellor [18]. Using electrical stimulation of the relevant neural circuitry, Panksepp and colleagues conceptualised and explored experimentally using electrical stimulation of the neural circuitry, a distinct set of emotional action systems adapted to guide the behaviour of mammals in the critical aspects of survival and reproduction, such as predator and disease avoidance, within-species competition, sexual partner choice and care of progeny. Seven basic emotional systems have been identified: RAGE/Anger, FEAR/Anxiety, and PANIC/Separation as negative-aversive emotional dispositions and LUST/Sexuality, CARE/Nurturance, PLAY/Joy and SEEKING/Expectancy as positive-appetitive dispositions [14,15,16]. Mellor and colleagues renamed the PANIC/Separation system as BONDING when considering positive effects because this system manifests two major types of motivation, separation distress and attachment rewards [18] and for the purpose of the present review, we will use the term BONDING for this system.

It is useful to consider which of these emotional dispositions are most likely to have effects on the behavioural responses of farm animals to humans. The FEAR system generates unequivocally negative emotions/affects that are characterised behaviourally as freezing or flight, while the emotional system of BONDING generates motivation for reunion. An example of BONDING is the separation distress expressed behaviourally by mammalian young that have been isolated after they have bonded with their mothers and the reduction in distress following reunion. In the case of response to humans, an example of BONDING may be a motivation to achieve or retain positive effects associated with social dependency and attachment [18]. The emotional system of PLAY motivates the animal to seek, explore and work to achieve the positive effects associated with the respective consummatory goals such as feeding, drinking, sexual activity, and social reinstatement [14]. The ‘SEEKING’ system supports expectancy, exploration, foraging, and other appetitive activities that are accompanied by positive effects. As concluded by Panksepp [14] and Mellor [18], the SEEKING system facilitates the goal-directedness of the positive effects associated with the BONDING and PLAY systems. Furthermore, the SEEKING disposition is specific in that it promotes active coping strategies through having effects on exploration, seeking and approaching specific sources of stimulation in the environment and the SEEKING disposition evokes feelings of positive excitement which control reward-learning [19].

It is this range of emotions/affects generated by the interaction with humans that is likely to determine an animal’s relationship with humans. As Waiblinger and colleagues [20] have suggested, different emotions and motivations are involved in the animal’s perception of and reaction to humans, and that these are likely to determine the extent of an animal’s relationship with humans, which will therefore vary from negative through neutral to positive. Furthermore, following Désiré and colleagues [11], the emotions of the animal expressed during interactions with humans are likely to be determined not only by the properties of the human in the relationship but also the animal’s interpretation of the whole situation, including the suddenness, familiarity, predictability, and capacity to control the interactions.

## 3. Fear, Stress, and Productivity: Interplay between HPA and HPG Axes

Studies on farm animals in both experimental and commercial conditions indicate that through learning, the history of the nature of previous stockperson interactions influences the animal’s current perception of humans, that is, the animal’s current relationship with humans [5,21,22]. For example, with handling of a predominantly negative nature, conditioned fear responses to humans may develop as a consequence of associations between the stockperson and the aversive elements of the handling bouts. Negative or aversive elements of handling from the animal’s perspective include abrupt and aggressive handling, and painful husbandry practices. While fear of humans can be reduced through habituation with repeated exposure to humans in a neutral context [21], there is also evidence that a positive relationship with humans can be established through a history of human contact that is predominantly rewarding in nature for the animal [21,22]. Rewarding elements of handling include feeding and human interactions such as talking and grooming depending on the species, as well as the opportunity for exploration (both inspective and inquisitive (curiosity) exploration), play, and companionship. A negative HAR from the perspective of the animal, therefore, can be defined operationally as the animal showing voluntary withdrawal and reduced spatial proximity (avoidance) and other behavioural indicators of fear such as agitation, vocalizations, orientation away, and posture, whereas a positive HAR from the perspective of the animal can be defined operationally as the animal voluntarily approaching and seeking spatial proximity and other expressions of seeking and pro-social or affiliative behaviours, such as anticipation, pleasure (e.g., playfulness and low-frequency vocalisations such as grunts in pigs) and relaxation (e.g., body, ear and tail posture depending on species and calmness) arising from interacting with the human [18,22].

As with behavioural responses, the physiological stress responses to fear-provoking stimuli can be varied and complex. Pioneer physiologists Cannon and Selye highlighted the non-specificity of the stress response. The physiological pathways of the stress response are highly conserved regardless of the nature of imbalance caused by a stressor, however, the magnitude of the stress response is determined by the extent of the imbalance to homeostasis caused by a stressor [23]. A cascade of events induced in response to stress generally includes activation of the autonomic nervous system, the neuroendocrine system consisting of the hypothalamo–pituitary–adrenal (HPA) axis, and the immune system [24,25]. The physiological pathways are integrated by the central nervous system and, in conjunction with behavioural responses, are utilised by the animal to cope when exposed to stressors. The sympathoadrenal system is activated within seconds of an animal becoming aware of a perceived or actual threat. Activation of this system results in secretion of noradrenaline (norepinephrine) neurotransmitters from postganglionic nerve terminals and adrenaline (epinephrine) neurotransmitters from preganglionic nerves of the adrenal medulla [26], and this, in turn, increases heart rate, blood pressure, and body temperature and mobilizes energy reserves by inducing adrenaline-dependent glucose secretion from the liver. This emergency reaction to the stressor lasts for a short period of time, and if the fear-provoking stimulus is not removed, a second type of defence involving the HPA axis, is activated. Stress-induced activation of the HPA axis causes the neurons of the paraventricular nucleus to release neuropeptides corticotropin-releasing hormone (CRH) and arginine vasopressin (AVP) in most mammals, except pigs, which secrete lysine vasopressin instead [27,28]. These neurohormones collectively have actions at corticotrophic cells in the anterior pituitary to induce the release of a range of peptides including adrenocorticotrophin (ACTH) [29,30] which induces the subsequent release of cortisol and other glucocorticoids from the adrenal cortex into peripheral circulation [31,32]. The activation of the sympathoadrenal system and the HPA axis is obviously an effective mechanism to assist the animal in adapting to changes in its environment. The physiological outcomes include circulatory and metabolic adjustments that allow the animal to react to physical and/or emotional challenges [5,23,24,33]. As part of the adaptive response, with acute stress, corticosteroids also suppress the inflammatory response, thereby reducing the detrimental effects of the immune response [5,23,24,33]. There are also some behavioural adaptations as a consequence of the short-term activation of the sympathoadrenal system and the HPA axis, such as increased arousal and alertness, and increased cognition, vigilance, and focused attention [5], that should contribute to the animal’s capacity to search, scrutinize and remember threatening or rewarding situations.

Thus, these transient biological responses are adaptive in nature and may contribute to the restoration of homeostasis and thereby improvement in the animal’s welfare state. While failure to adapt may ultimately result in death [33], less severe challenges can result in fewer biological costs, such as impaired growth [34], health, and reproduction [35,36].

**Figure 1:**
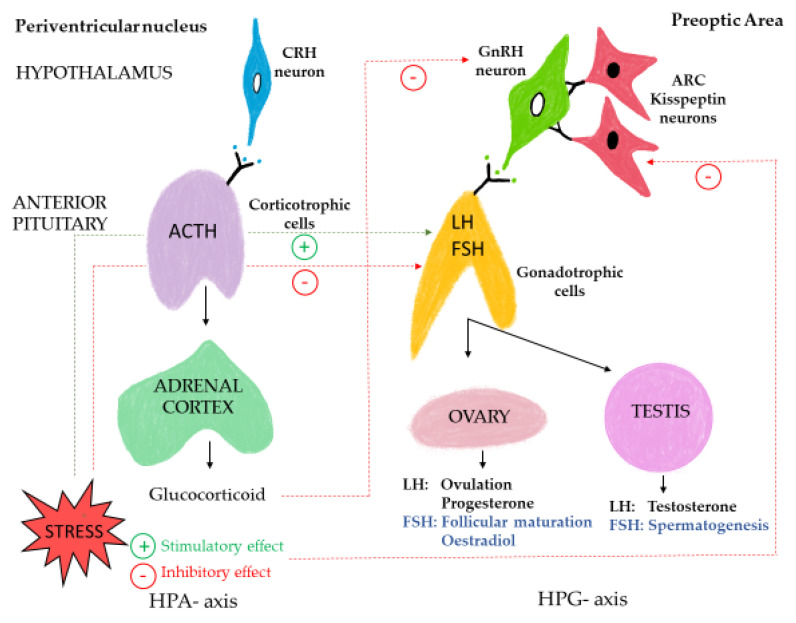
Potential sites along the HPG axis which may be influenced by stress-induced activation of the HPA axis (adapted from [41]).

Reproduction is an imperative biological function, and it requires considerable stress to suppress it [23]. Prolonged stress, however, has been reported to impair reproduction in many species of animals, for example, pigs [37,38,39,40,41] and laying hens [42]. The scientific literature on the direct effects of human-animal interactions on the reproductive functions of animals, however, is lacking. Some of the potential physiological pathways by which the HPA axis influences the hypothalamic-pituitary gonadal (HPG) axis are shown in Figure 1 (Adapted from Tilbrook and colleague [41]). It is generally accepted that there is a reciprocal association between the HPA axis and the HPG axis functions, whereby activation of one of these axes has effects on the activation of the other axis [43]. Hence, the main focus in this section of the review will be on the potential effects of HAR on the HPG axis when there are effects as a result of activation of the HPA axis. Central to the HPG axis is the episodic secretion of gonadotrophin-releasing hormone (GnRH) by a unique heterogenous population of neurons located in the mediobasal septum and preoptic area of the hypothalamus. Gonadotrophin-releasing hormone (GnRH) is the primary hypothalamic factor regulating pituitary gonadotrophin secretion and overall reproductive functions [44,45]. The GnRH decapeptide is transported via the venous plexus to the anterior pituitary gland where it binds to specific receptors of gonadotrophs. The binding of GnRH to gonadotrophic cells stimulates the synthesis and release of luteinizing hormone (LH) and/or follicle-stimulating hormone (FSH) into the vessels of the secondary portal plexus [46,47]. These gonadotropins are subsequently transported to the gonads where LH and FSH bind to specific protein receptors, with the consequent synthesis and secretion of gonadal steroid hormones such as oestrogen and progesterone from the ovaries and testosterone from the testis [48]. There are receptors for gonadal steroids in many reproductive tissues of the body where binding of these steroids to the receptors induces physiological responses that are faciliatory in inducing reproductive behaviours, gamete maturation in both the ovaries and testes, transport of sperm during the ejaculatory processes, transport of gametes to the site of fertilization in females, and processes that are essential during pregnancy and the gestational period. When there are external factors, such as stress that disrupt the functions of the HPG axis, reproductive processes can be compromised. The functions and pathways of the HPA axis are similar to those of the HPG axis, however, the signalling hormone produced by activation of the HPA axis is CRH rather than GnRH. The CRH is transported into the same tissues and through the same vascular systems as GnRH, however, due to the unique chemical structure of CRH, it binds to the corticotrophs in the anterior pituitary inducing the release of the previously synthesized adrenocorticotropic hormone (ACTH). The ACTH is transported via the bloodstream to the adrenal cortex where it has effects on steroidogenic pathways to induce the release of cortisol and some other glucocorticoids, particularly corticosterone in some animals, which modulate many physiological responses to stress. Prolonged fear-provoking stimuli can result in sustained activation of the HPA axis, which subsequently leads to a reproductive stress response by inhibiting the function of various processes of the HPG axis [49,50]. Sustained secretion of CRH and beta-endorphins may inhibit the synthesis and/or secretion of GnRH from the hypothalamus [32]. Additionally, greater concentrations of glucocorticoids induced by prolonged or chronic stress suppress the activity of the kisspeptin-producing neurons, which control episodic releases of GnRH from the hypothalamus [51] and, therefore, inhibit functions of the downstream components of the HPG axis. Inhibitory effects of glucocorticoids and prolactin can also reduce the responsiveness of the pituitary to GnRH, thereby reducing the secretion of LH and FSH. Chronic increases in concentrations of glucocorticoids also reduce ovarian sensitivity to LH by inhibiting the secretion of oestrogen and progesterone. Glucocorticoids also reduce testicular sensitivity to GnRH resulting in decreased sperm production [23]. As described earlier in this review, the magnitude and duration of the activation of the HPA axis are determined by the aversiveness and the duration of the stressor, making it difficult to study the downstream effects of the stress response in experimental settings. The short-term physiological changes resulting from acute stress may be an adaptive response with minimal biological costs, which is inconsistent with what occurs when there is chronic stress (i.e., sustained increase in glucocorticoid concentrations) which may induce profound downstream effects on gonadotropins and hence the overall reproductive function of an animal. In the subsequent sections of this manuscript, we explore studies where results indicate there are differing effects of human-animal interactions on reproductive status of the animals.

Obviously, while biological regulation in response to stressors occurs continuously, adaptation is not always possible [5,52]. Indeed, marked perturbations to homeostasis may overwhelm an individual’s capacity to adapt and lead to its death. Nevertheless, less severe perturbations can still have marked effects on biological systems, leading to growth, reproductive, health, and other impairments, which may reflect and/or result in welfare problems for the animal [24,33].

### 3.1. Evidence from Experimental Studies

In most studies in which the effects of stress on reproduction have been examined, stress has been induced by administering neuroendocrine compounds directly into animals to mimic the activation of a stress response [53,54]. Extrapolating the effects of stress from laboratory experiments to animals in captivity and that are resident in their natural habitats should occur with caution. The administration of neuroendocrine compounds directly into animals may not induce the same cascade of physiological responses or magnitude of physiological effects as compared with stressors in captive animal settings, such as livestock production.

Results from the experimental studies, predominantly with pigs, dairy cattle, and poultry, indicate that negative or aversive handling imposed briefly but regularly increases fear of humans in conjunction with a sustained increase in the glucocorticoid concentrations [5,13]. For example, imposition of a slap, hit, or shock with a battery-operated prodder whenever pigs approached or failed to avoid an experimenter during daily brief handling bouts consistently resulted in increased fear of humans, based on approach behaviour to an unfamiliar human, in comparison to imposing a pat or stroke whenever pigs approached the experimenter [34,35,55,56,57]. In many of these experiments, brief negative handling increased both the acute cortisol response to humans and basal cortisol concentrations and reduced growth rate [34,35,58]. For example, Hemsworth and colleagues [58] reported an increase in cortisol concentrations in gilts subjected to brief daily negative handling that was sustained beyond the period of treatment imposition. Turner and colleagues found that a sustained increase in cortisol during the oestrous cycle was needed to inhibit the preovulatory release of LH, rather than a repeated acute increase in cortisol [54]. These findings along with other similar studies highlight that the circulating concentrations of cortisol need to be increased beyond basal for a substantial period of time to impair reproductive processes in the female pig [53,54,59]. Brief negative handling leads to a reduced testicle size in boars at 23 weeks of age and delay in the age of a coordinated mating response [35]. In a short-term experiment in gilts, brief daily negative handling imposed 8 d prior to expected behavioural oestrus increased cortisol concentrations for at least 3 to 4 h after each individual handling bout. The daily negative handling, however, did not affect the reproductive parameters such as sexual behaviour, pregnancy rate, and number and weight of embryos 20 to 21 d after insemination [60]. Hemsworth and colleagues [35] found that brief negative handling of gilts led to a reduction in pregnancy rate, without having an effect on their sexual behaviour when mated with non-experimental boars.

It is likely that short-term imposition of repeated acute stressful procedures, such as negative handling, might affect the synthesis in and/or secretion from the hypothalamus of GnRH without having any effects on LH and FSH production by the anterior pituitary glands. There is a threshold abundance of kisspeptin-1 mRNA transcript that is thought to be necessary for GnRH synthesis to occur in both the medial-basal hypothalamus and preoptic area before there is any detectable reduction in GnRH mRNA transcript and subsequent LH secretion. The lack of direct effects when there is stimulation of the HPG axis on gonadotropin release from the anterior pituitary even though there are effects on GnRH synthesis could be attributed to the reserves of gonadotropin subunits in the anterior pituitary, which can result in the secretion of LH and FSH independent of GnRH secretion from the hypothalamus. Pigs that are highly fearful of humans with a history of negative human interactions, not only have an acute cortisol response in presence of humans but may also have a sustained increase in the basal cortisol concentrations. Results from a number of studies indicate that a sustained increase in glucocorticoids relative to the basal concentrations in pigs, results in a reduction of circulating concentrations of LH [53,54,59], and therefore, the expectation would be that gonadal functions regulated by LH would be compromised when there is chronic or sustained stress.

There is limited research on the direct effects of positive handling on reproductive performance in pigs. Anderson and colleagues [61] reported that positive handling of sows in the latter stages of the gestation did not affect the reproductive outcome, as indicated by piglet mortality or early lactation piglet weight gain, or have any effect on maternal behaviour post-farrowing. Similarly, Hayes and colleagues [62] reported there were no effects of daily brief positive handling during gestation on the reproductive performance of sows as assessed by the number of piglets born alive, the number of stillborn piglets, piglet growth, and the number of piglets weaned. While the sample size was small, in the study by Hemsworth and colleagues [35] pregnancy rate in gilts handled negatively from an early age was less than that of gilts handled positively, with an intermediate pregnancy rate for gilts that received minimal human contact.

There is also evidence from other farm animal species that negative handling has both productivity and welfare consequences. For example, results from handling studies with dairy cattle indicate negative handling may suppress milk yield in cows [63,64,65]. Rushen and colleagues [63] reported that dairy cows had larger quantities of residual milk when milked in the presence of an experimenter that had previously handled the cows in a negative manner than in the presence of another experimenter that had positively handled the cows. In the presence of the negative handler, the cows also had greater heart rates at the time of milking, implicating a greater secretion of catecholamines as a result of activation of the autonomic nervous system leading to inhibition of milk release from the alveoli of fearful cows. Lactation failure and accumulation of residual milk in cows when in close proximity to a handler who has previously imposed negative handling could either be due to stress-induced inhibition of oxytocin secretion [65,66] or secretion of catecholamines as a result of effects at the autonomic nervous system [63]. Impaired oxytocin release negatively affects the contraction of myoepithelial cells surrounding alveoli in mammary glands, which in turn leads to an inhibition of milk release from the alveoli and, as a consequence, an increase in residual milk in the mammary glands after completion of milking. Neumann and colleagues [67] reported in lactating rats there was a stress-induced auto-inhibition of oxytocin secretion from the posterior pituitary that resulted in reduced oxytocin concentrations in blood as well as a delayed milk release from the alveoli and suppression of lactational processes. This could explain why there is delayed milk ejection and accumulation of residual milk when cows are milked in the presence of a handler with a history of negative handling. Interestingly, this autoinhibitory effect of oxytocin is only observed during the peripartum period, in particular during late pregnancy and lactation in rats. In non-pregnant animals, oxytocin release, oxytocin receptor quantities, and binding in many regions in the brain are involved in the regulation of activity of CRH neurons and thereby the induction of the negative feedback effects on the HPA axis [68].

Human contact affects reproduction in laying hens. Hughes and Black [69] reported that handling of laying hens unaccustomed to handling had less egg production. Barnett and colleagues [70] reported that regular visual contact, involving positive elements such as slow and deliberate movements by humans, resulted in greater egg production than a treatment that involved minimal human contact that at times contained elements of sudden, unexpected human contact. The hens in the minimal treatment had greater avoidance and plasma corticosterone responses in the presence of humans and the authors speculated that the lesser egg production of these hens may be a consequence of a chronic stress response because there was evidence of immunosuppression in these hens. There is evidence that a prolonged increase in adrenaline concentrations can affect egg productivity by three different mechanisms; inhibiting ovulation, inducing oviduct immobility resulting in internal egg-laying, or altered muscular contraction in shell glands resulting in eggshell deformation [70]. A prolonged increase in adrenaline concentrations can affect egg productivity by three different mechanisms; inhibiting ovulation, inducing oviduct immobility resulting in internal egg-laying, or altered muscular contraction in shell glands resulting in eggshell deformation [71].

As described previously in this review, farm animals may have positive emotional experiences in the presence of humans that may arise from associating humans with rewarding events, such as feeding, patting, and grooming. This may subsequently result in attraction to humans on the basis of approach behaviour. For example, stroking applied to cattle in a manner that is similar to intraspecific allogrooming leads to a reduced heart rate and results in relaxed body postures and increased approach to humans (see reviews [5,21,22]). Animals that have positive emotional experiences in the presence of humans may also have reduced stress responses in unfamiliar or painful situations, such as veterinary inspections or husbandry interventions or even when housed in suboptimal environments. Brief daily positive human contact leads to a lesser magnitude of the physiological stress response to tether housing of sows [72]. Although a positive HAR can have positive welfare outcomes for farm animals, there is little evidence of the benefits of a positive HAR on farm animal reproduction.

### 3.2. Evidence from On-Farm Studies

In parallel with the experiments examining the effects of handling on livestock (Section 3.1), results from field studies indicate that there are sequential relationships between stockperson characteristics, particularly attitudes and behaviour in relation to handling farm animals, and fear of humans and productivity of farm animals. There were three important findings from these field studies. Firstly, there were negative inter-farm correlations between fear of humans and productivity. Fear was assessed on the basis of the animal’s behavioural response to humans, and the productivity variables associated with fear of humans were the number of piglets produced per sow per year in pigs [58,73], milk yield in dairy cows [64,74], egg production in laying hens [42,75] and feed conversion efficiency and mortality of meat chickens [76,77]. The extent of fear of humans was also positively correlated with increased percentage of stillborn piglets in commercial sows [78]. Secondly, there were inter-farm correlations between the nature of the stockperson behaviour and fear of humans in pigs and dairy cows [64,73,74,79]: increased use of negative behaviours such as slaps, hits, and/or fast speed or sudden movement and less use of positive behaviours such as pats, strokes, talking and /or slow movement was associated with a greater fear of humans. There were some similar relationships in poultry. Results from two studies with commercial meat chickens indicated that the speed of movement by the stockperson was correlated positively with the avoidance of meat chickens to an approaching experimenter [77,80], while Edwards and colleagues [75] reported that the duration of peak egg production was negatively associated with both the noise made by the stockperson and hen fear of humans. Thirdly, there were inter-farm correlations between stockperson attitudes, assessed on the basis of their beliefs about handling and working with their animals, and stockperson behaviour. In general, positive attitudes to the use of petting (patting and talking) and the use of verbal and physical effort to handle dairy cows and pigs were associated with less use of negative behaviours when working with dairy cows and pigs [58,64,73,74,81], while negative attitudes to the sensitivity of laying hens to human contact, as well as negative general beliefs about hens, were associated with more noise, faster speed of movement and less time stationary near hens by stockpeople [75].

The causality of these sequential relationships observed in the field has been demonstrated in field experiments in the pig and dairy industries. Results from randomised design intervention studies in these industries indicated cognitive behavioural training, in which the key attitudes and behaviour of stockpeople are targeted, improves the attitudes and behaviour of stockpeople towards their animals, with consequent beneficial effects on animal fear and productivity (for example, see Table 1).

**Figure 2:**
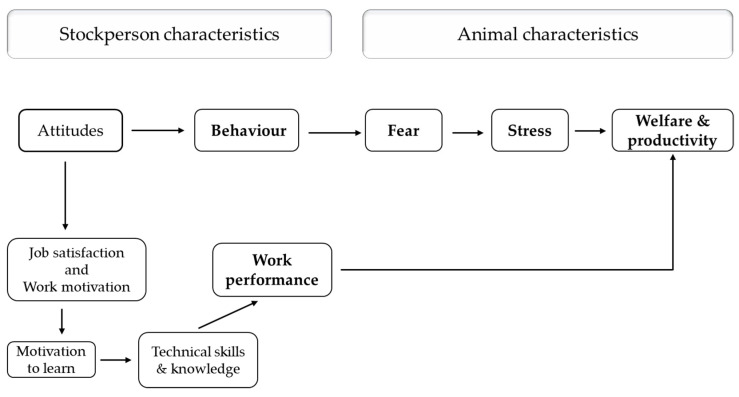
Sequential relationship between stockperson attitudes, behaviour and job-related characteristics and fear, stress, and productivity in farm animals (adapted from [83]).

In addition to the direct effects of stockperson behaviour on animal fear, welfare and productivity, there may be indirect effects of stockperson attitude and job-related characteristics on the above-mentioned animal variables. As seen in Figure 2, the attitude of the stockpeople towards animals in their care may affect their job satisfaction, work motivation, and the motivation to learn new skills about animals, which may collectively affect their work performance. Indeed results from a study with pig stockpeople showed that the stockperson’s willingness to attend training sessions in their own time was correlated with their attitude towards specific characteristics of pigs and most aspects of their work [82].

Both direct and indirect relationships between stockperson attitude, behaviour, and work performance on animal welfare and productivity highlight the importance of training that targets specific attitudes and behaviours of stockperson towards farm animals. Such training can be provided using approaches such as cognitive-behavioural techniques. Details of these cognitive-behavioural techniques and the rationale for their use to improve stockperson attitudes and behaviour have been described in detail by Hemsworth and Coleman [5] and Coleman and Hemsworth [10]. Cognitive-behavioural techniques basically involve changing a person’s behaviour by first targeting both the beliefs that underlie the behaviour (attitude) and the behaviour in question, and second, maintaining these changed beliefs and behaviours. This process of inducing behavioural change is a comprehensive procedure in which all personal and external factors that are relevant to the behavioural situation are explicitly addressed. This includes addressing commonly perceived barriers to change, addressing defensiveness about previous behaviour, changing habits, providing follow-up sessions to reinforce changes as well as changing the relevant attitudes and behaviour.

**Table 1:** Effects of cognitive behavioural training of dairy stockpeople (adapted from [84,85]).

Variables	Change Following Training (Relative to Control)	*p* Value
*Stockperson attitudes*		
+ve Beliefs about ‘effort’	16% ↑	0.001
+ve Beliefs about ‘petting’	25% ↑	0.01
*Stockperson behaviour*		
−ve behaviour	50% ↓	0.001
*Cow behaviour* Flight distance (m)	7% ↓	0.05
*Cow physiology* Milk cortisol (nM/L)	32% ↓	0.06
*Cow productivity* Milk yield (L/cow/month)	5% ↑	0.02

Thus, the skills, knowledge, and motivation of stockpeople to effectively care for and manage their animals are integral to both the welfare and productivity of farm animals. Attitudes of stockpeople influence not only the manner in which stockpeople handle their animals, but also their motivation to care for their animals [5]. Training targeting technical skills and knowledge, as well as the attitudes and behaviours of stockpeople, therefore, should be a primary component of the human resource management practices at a farm.

## 4. Conclusions

There is considerable evidence that the history of human interactions leads to a stimulus-specific response of farm animals to humans. The animal’s perception of humans in turn has implications for its welfare, as emotions that are generated in the farm animals following interactions with humans directly affect the animal’s welfare. Repeated negative interactions by stockpeople can lead to greater fear of humans in farm animals, which in turn through stress can compromise reproductive efficiency in these animals. Understanding stockperson–farm animal relationships have implications for improving farm animal welfare and productivity, including reproductive efficiency. Stockperson attitudes are amenable to change, and hence stockperson training can improve human–animal relationships in the livestock industries. Our understanding of the implications of a positive perception of humans on animal welfare and productivity, including reproductive outcomes, is less clear and thus this is a topic that requires research.

## Data Availability

Not applicable.
